# Longitudinal study assessing the return of chloroquine susceptibility of *Plasmodiun falciparum* isolates from travelers returning from West Africa

**DOI:** 10.1186/1475-2875-11-S1-P38

**Published:** 2012-10-15

**Authors:** Myriam Gharbi, Jennifer Flegg, Véronique Hubert, Eric Kendjo, Jessica Metcalf, Philippe J Guerin, Jacques Le Bras

**Affiliations:** 1 UMR2I6: Mère et enfant face aux infections tropicales, Université Paris Descartes-Paris 5 and Institut de Recherche pour le Développement (IRD), Paris, France; 2 WorldWide Antimalarial Resistance Network (WWARN), Paris, France; 3 Ecole des Hautes Etudes en Santé Publigue (EHESP), Rennes, France; 4 WorldWide Antimalarial Resistance Network (WWARN), Oxford, UK; 5 Centre for tropical Medicine, Nuffield Department of Clinical Medicine, CCVTM, University of Oxford, Oxford, UK; 6 Centre National de Référence du Paludisme, Paris, France; 7 Service de Parasitologie Mycologie, CHU Bichat-Claude Bernard, APHP, Paris, France; 8 Service de Parasitologie Mycologie, CHU Pitié-Salpétrière, APHP, Paris, France; 9 Epidemiology and Infectious Diseases, Department of Zoology, University of Oxford, Oxford, UK; 10 UMR S 707: Epidemiology, Information Systems, Modeling, INSERM and Université Pierre et Marie-Curie-Paris, Paris, France

## Background

From the 1940s up to the 1990s, chloroquine (CQ) was the main malaria therapy worldwide. Following the CQ resistance burden in Africa, most African countries have discontinued CQ during the past 2 decades, and now promote artemisinin-based combination therapy (ACT), as the first-line treatment for uncomplicated malaria. The policy changed in West Africa during the last decade (2002 in Cameroon; 2003 in Senegal and Cote d’l voire; 2004 in Mali). The aim of this study is to describe the evolution of CQ resistance in West Africa, through travellers returning from this region.

## Methods

The study was conducted by the Malaria National Reference Centre, France. The database collated *in vitro* response of reference and clinical isolates for CQ and the *pfcrtK76* molecular marker for CQ susceptible *Pf* malaria from travellers returning from Cameroon, Senegal, Cote d’lvoire and Mali. As a proxy of drug pressure, CQ intake for children under five years of age with fever was extracted from the Demographic Health Surveys (DHS) and Multiple Indicator Cluster Surveys (MICS) for the study period [1,2]. Logistic regression models were used to detect trends in the susceptible isolates proportions.

## Results

From 2000 to 2011, around 700 isolates were genotyped for each country. The frequency of the *pfcrt76* wild-type significantly increased for Cameroon (CM) (from 10% to 41%, Slope=0.09, p<10-3), Cote d’lvoire (CI) (from 37% to 63%, Slope = 0.14, p<10-3), and Senegal (SN) (from 22% to 53%, Slope=0.17, p<10-3). The geometric mean of the 50% growth inhibition (IC50) of CQ decreased from 181 nM (95% confidence interval, 87-374) (25% CQ sensitive) to 51 nM (37-71) (63% CQ sensitive) in CM, from 75nM (43-130) (41% CQ sensitive) to 29nM (22-39) (84% CQ sensitive) in CI and from 86nM (51-145) (41% CQ sensitive) to 39nM (26-60) (75% CQ sensitive) in SN. Analyses performed from 2004 to 2011, when most of West African countries have officially discontinued CQ, confirmed previous results and also show a significant increase of the prevalence of pfcrt76 wild-type genotype for Mali (ML) (Slope = 0.07, p=0.02). Meanwhile, CQ use among children with fever significantly decreased during this period.

## Conclusions

An increase of CQ susceptibility following official withdrawal is observed in travellers returning from Cameroon, Cote d’lvoire, Mali and Senegal. The length of time between policy changes and their subsequent implementation, as well as the cross resistance between antimalarial drugs, may affect the time for a significant recovery of CQ sensitivity. This information should be compared to country level CQ efficacy data.
